# Implementation, coverage and equity of large-scale door-to-door delivery of Seasonal Malaria Chemoprevention (SMC) to children under 10 in Senegal

**DOI:** 10.1038/s41598-018-23878-2

**Published:** 2018-04-03

**Authors:** El-Hadj Bâ, Catherine Pitt, Yankhoba Dial, Sylvain Landry Faye, Matt Cairns, Ernest Faye, Mouhamed Ndiaye, Jules-Francois Gomis, Babacar Faye, Jean Louis Ndiaye, Cheikh Sokhna, Oumar Gaye, Badara Cissé, Paul Milligan

**Affiliations:** 1 Institut de Recherche pour le Développement, Dakar, Senegal; 2 London School of Hygiene & Tropical Medicine, London, UK; 3 Ministère de la Santé et de la Prévention, Dakar, Senegal; 4 Université Cheikh Anta Diop, Dakar, Senegal

## Abstract

SMC has been introduced widely in the Sahel since its recommendation by WHO in 2012. This study, which provided evidence of feasibility that supported the recommendation, included school-age and pre-school children. School-age children were not included in the 2012 recommendation but bear an increasing proportion of cases. In 2006, consultations with health-staff were held to choose delivery methods. The preferred approach, door-to-door with the first daily-dose supervised by a community-health-worker (CHW), was piloted and subsequently evaluated on a large-scale in under-5’s in 2008 and then in under-10’s 2009–2010. Coverage was higher among school-age children (96%(95%CI 94%,98%) received three treatments in 2010) than among under 5’s (90%(86%,94%)). SMC was more equitable than LLINs (odds-ratio for increase in coverage for a one-level rise in socioeconomic-ranking (a 5-point scale), was 1.1 (0.95,1.2) in 2009, compared with OR 1.3 (1.2,1.5) for sleeping under an LLIN. Effective communication was important in achieving high levels of uptake. Continued training and supervision were needed to ensure CHWs adhered to treatment guidelines. SMC door-to-door can, if carefully supervised, achieve high equitable coverage and high-quality delivery. SMC programmes can be adapted to include school-age children, a neglected group that bears a substantial burden of malaria.

## Introduction

Since 2012, countries in the Sahel have started to introduce Seasonal Malaria Chemoprevention (SMC) for children under 5 years of age. The current policy is limited to children under the age of 5 but in many parts of the Sahel there is a substantial burden of malaria in older children who could benefit from SMC. SMC involves monthly antimalarial treatment for up to four months to prevent malaria, and is recommended by the World Health Organization (WHO) for children who live in areas with intense and highly seasonal malaria transmission in the Sahel sub-region^1^. In 2017, twelve countries had SMC programmes reaching about 15million children (Burkina Faso, Cameroon, Chad, Gambia, Ghana, Guinea, Guinea Bissau, Mali, Niger, Nigeria, Senegal and Togo). In Senegal, children up to 10 years of age are included, while in all other countries SMC is limited to children under 5 years of age in accordance with the current WHO recommendation.

Prior to WHO’s recommendation, key concerns regarding SMC were the feasibility of delivery on a large scale through routine health services and the most appropriate delivery methods, especially given the requirement that SMC be delivered at monthly intervals during the rainy season. Preliminary findings from the study reported here contributed to WHO’s 2012 policy recommendation. The possibility of extending the recommended age range to include children up to the age of 10 is now also under consideration.

Several possible delivery methods for SMC were considered for children under 5. In The Gambia, a cluster-randomized trial in 12,000 children found that village health workers achieved substantially higher coverage than delivery through mobile, nurse-led vaccination clinics, and that delivery by village health workers was both equitable and more cost-effective^2^. In Ghana, a small village-randomized trial found that community volunteers achieved slightly higher coverage than health workers in clinics and outreach clinics, although both achieved high coverage; they concluded that a combination of approaches could be necessary in some settings^3^. In Mali and Burkina Faso, a qualitative study found that parents and community health workers (CHWs) involved in a clinical trial of SMC efficacy, largely favoured future SMC distribution from fixed points in villages, the method used in the trial, and were concerned about the ability of families to administer the second and third daily doses of amodiaquine (AQ) at home^4^. The studies in Ghana and The Gambia included relatively small numbers of children in a single year with substantial implementation support from researchers, while the efficacy trials in Mali and Burkina Faso employed researcher-led distribution strategies. Implementation research in an operational setting was therefore important to address questions as to whether and how routine health services could successfully deliver SMC on a large scale and achieve high and equitable coverage, and about how to reach older children. In Senegal, consultations were held with health staff to select the most appropriate method of delivery. This method was piloted, and subsequently evaluated on a large scale in a range of settings in children under 5 and then in children up to 10 years of age (children aged at least 3 months and who, at first monthly treatment of the year, are less than 10 years old. Children who reach 10 years of age during SMC continue to receive treatments to the end of the transmission season but are not eligible the following year). In this paper, the findings from the pilot study, and the results on SMC delivery from the large-scale study, are presented. The primary results from the large-scale study have been published separately^5^.

### Study Overview

We report on the process of developing and adapting the methods for implementing SMC in central Senegal over a 5-year scale-up period and the levels of coverage and equity achieved. A randomized trial in Niakhar, Senegal^6^ had shown that monthly administration of antimalarial treatment (a one-day regimen of sulfadoxine plus one dose of artesunate) to children resulted in an unprecedented level of protection, with an efficacy of 86%. A subsequent trial^7^ showed that sulfadoxine-pyrimethamine plus amodiaquine (SP + AQ) was an even more effective regimen. Use of SP + AQ required a 3-day course of treatment each month, but had the advantage, in addition to improved efficacy, that its use for SMC would allow artemisinin combinations to be reserved for treatment of acute malaria cases, where their rapid action would be most beneficial. Implementation research to determine how SMC with SP + AQ could be delivered most effectively was then an urgent priority. In 2006 and 2007, a pilot study was conducted to develop practical methods for implementation suitable for the local health system context; SMC was delivered in the catchment areas of two rural and one semi-urban health post, while one rural health post was observed as a control in 2006 and began implementation from 2007. Over the following three malaria seasons (2008–10), SMC was adapted and rolled out in a stepped wedge randomized implementation trial, reaching 9 rural and semi-urban health posts’ catchment areas in 2008, 27 in 2009, and more than 150,000 children across 46 health posts in 2010 (Table 1). Of the 78 health posts in the three main implementation districts, 9 rural and semi-urban health posts were observed as controls throughout the study period and 23 urban health posts were excluded, as operational conditions and epidemiology were expected to differ substantially in urban settings. Further details of the design and randomization process are provided elsewhere^5^.

**Table 1:** Implementation summary.

Year (phase)	Target age group	District	Rural or semi-urban	Health posts implementing SMC	CHWs	Children^1^ per CHW (mean)	Children^1^ per CHW per day (mean)
2006 (pilot)	2–59 months	Cherif Lo Health Post	Rural	1	35	65	22
		Medine Health Post	Semi-urban	1	21	70	23
		Pambal Health Post	Rural	1	20	96	32
		Total		3	76	74	25
2008 (main study)	3–59 months	Bambey District	Rural	3	50	87	29
		Fatick District	Rural	3	68	73	24
		Mbour District	Semi-urban	3	77	81	27
		Total		9	195	80	27
2009 (main study)	3–119 months	Bambey District	Rural	9	254	155	39
		Fatick District	Rural	10	178	166	42
		Mbour District	Semi-urban	8	153	138	34
		Total		27	585	154	39
2010 (main study)	3–119 months	Bambey District	Rural	16	380	199	51
		Fatick and Niakhar Districts^2^	Rural	17	272	181	45
		Mbour District	Semi-urban	13	238	179	37
		Total		46	890	186	44

^1^Includes doses that were refused or rejected.

^2^In 2010, Fatick was officially divided into two districts: Fatick and Niakhar. For comparability, they are listed together for all three years.

For the pilot study and the first year of large-scale implementation, SMC was delivered to children under 5 (aged 3 to 59 months), however, in response to the changing malaria epidemiology, the target age range was expanded in 2009 and 2010 to children under 10 (aged 3 to 119 months).

**Figure 1:**
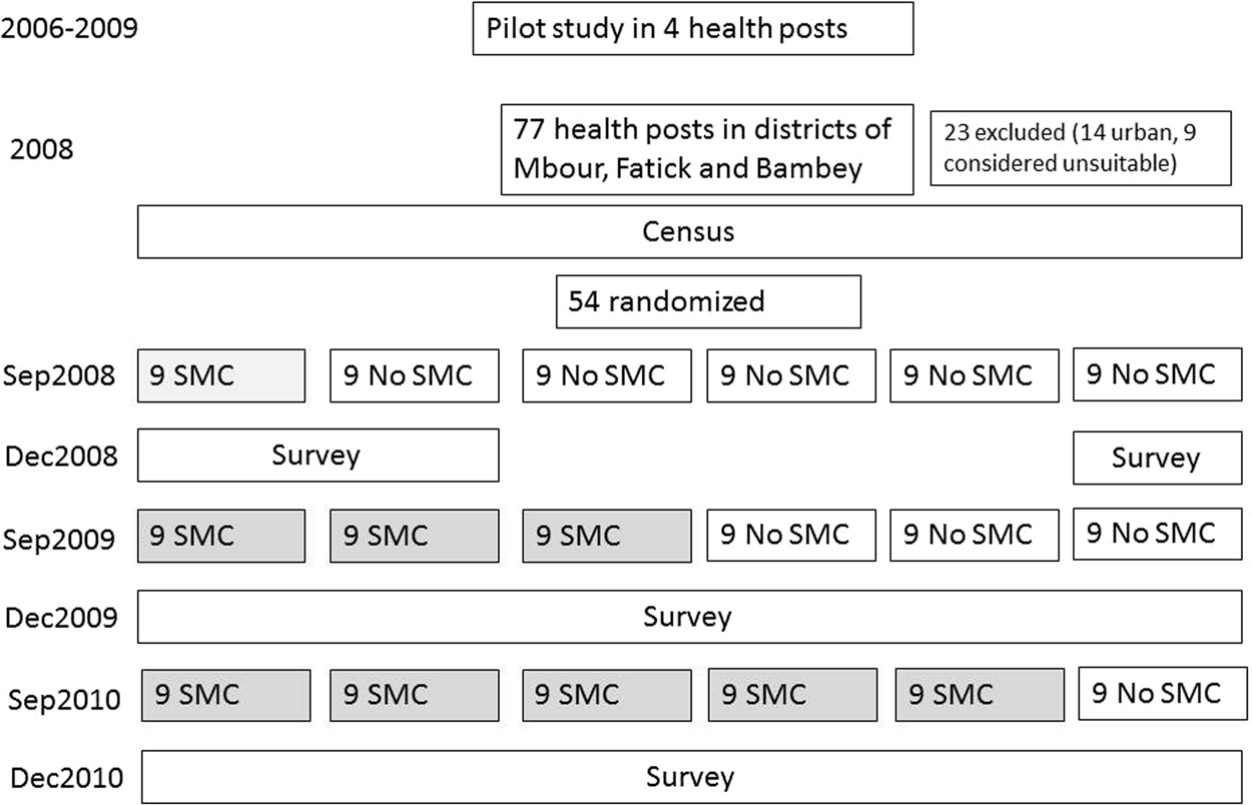
Study profile.

In the following sections, we describe the setting and then report on the pilot study, followed by the main implementation trial, with the aim of informing ongoing implementation across the Sahel and further policy development. The study profile is shown in Fig. 1.

### Setting

Tivaouane District (Thiès Region) was selected for the pilot because it included both rural and semi-urban health posts and, when the study was planned, suffered high incidence of malaria. The main implementation trial was conducted in the three neighbouring districts of Mbour (Thiès Region), Bambey (Diourbel Region), and Fatick (Fatick Region), which shared similar health and socio-demographic characteristics to Tivaouane. In 2010, Niakhar District was created from part of Fatick District and continued to be included in the implementation area. The study population is described by Cisse *et al*.^5^.

In 2010, the 46 health posts delivering SMC in the main study area served a median population (all ages) of 10,236 people (range: 1,943 to 45,261) per health post. Each health post is normally staffed by a full-time head nurse and three support staff, all considered CHWs: a community health agent (*agent de santé communautaire*, ASC) acting as an assistant nurse, a CHW (*relais*) responsible for taking payments, and a birth attendant (*matrone*); in some posts, a midwife is also employed.

In Senegal, several categories of CHW are involved in primary health care programmes. Community health agents or *agents de santé communautaire* (ASC) are literate, lay health workers who have received specific training and perform auxiliary roles, including basic curative care, within the health post or health hut. By contrast, *relais* are more numerous and expected to be a key link between the health post and local communities; their qualifications vary and the head nurse calls on them to support the health post in information, education, and communication and in mass distribution campaigns, which the ASCs also support. *Relais polyvalents* mainly support the expanded programme on immunization, but also contribute to HIV/AIDS, malaria, tuberculosis, nutrition, and water and sanitation activities. Both the ASCs and *relais polyvalents* in the study area are members of associations, which perform functions similar to labour unions. Certain other *relais* work on a single programme, notably nutrition. ASC’s are paid a monthly salary by the Comité de Santé, a local committee that oversees the work of the health post and decides how resources are used. *Relais* are paid on a daily basis through the district health office.

For large campaigns, including SMC, additional literate community members, such as secondary school students, may also be called on to act as *relais* on a temporary basis. Twice per year (usually June and December) CHWs deliver Vitamin A and mebendazole to children under 5 year through door-to-door visits. In Bambey district, from 2006 to 2009, CHWs also delivered azithromycin to treat trachoma to all residents aged over 6 months except pregnant women each year in November - December. We use the umbrella term “CHW” throughout the paper to refer to both the small number of ASCs, the much larger number of *relais*, and additional community members acting as temporary *relais*.

## Pilot Study

### Pilot methods

#### Developing the approach to delivery

**Consultation process:** The delivery approach was developed through a district-level consultation process. The district medical officer (YD) chaired group discussions with the district public health officer, district health communications officer, the head nurses of the four health posts involved in the pilot, leaders of two CHW unions, staff of the Service de Lutte Anti-Parasitaire (SLAP), and members of the research team (including PM, BC, EF, SF). The research team presented the intervention approach, which called for children to receive a therapeutic course of SP + AQ each month for three months of the malaria transmission season (September-November). The need to ensure strict timing, accurate dosing, good adherence, and safe drug use were highlighted.

Delivery of SMC door-to-door, at fixed points in each village, and at health facilities, were considered. Supervised administration of all three daily doses, or of the first dose only, each month, and the possibility of leaving treatment drugs to be administered unsupervised, were also examined. How best to document SMC doses - on the existing health card or on an SMC record card – was also debated. Likely implementation challenges and options were discussed, and experiences shared of successful door-to-door CHW campaigns for Vitamin A, deworming, vaccination, azithromycin for trachoma, and other programmes. The research team intended that the three health posts would each implement their chosen approach in the pilot study to identify the most successful strategies. The head nurses, however, all agreed that CHWs should deliver SMC door-to-door. Key factors in this choice were the positive experiences of door to door delivery of other interventions; concerns, based on previous experience, that attendance at fixed points for a preventive intervention would be poor; and because it was felt that CHWs needed time to explain the nature and purpose of the intervention to each family to ensure acceptability and adherence and to avoid misconceptions and spread of rumours about drug side effects. CHWs delivering these messages should be individuals known and trusted in the community. It was felt that if this approach was adopted, the tablets for days 2 and 3 could be left with the caregiver who could be relied upon to ensure that the course of treatment was completed without the need for supervision. A door-to-door strategy was therefore adopted by all three health posts. It was agreed that effective community sensitisation would be needed to explain the programme and to publicise dates of campaigns. It was considered important that families would have a record of the treatments their children received, so an SMC card was devised. The importance of using rapid tests to diagnose malaria in children attending clinic with fever (as opposed to treating presumptively as if malaria) was also noted, since in children who had received SMC, fever was less likely to be due to malaria.

**Eligibility**, **Drug dosage and formulation**, **and administration:** For the pilot studies, all children aged 2 to 59 months who were normally resident in the study area were considered eligible unless they had a fever (in which case they should be referred to the nearest health structure where cases of fever were tested for malaria), or had a history of allergy to SP or AQ. Dosage of AQ and SP should, ideally, be based on a child’s weight, however, this is often impractical and especially in door-to-door campaigns. Anthropometric survey data (K Simondon, unpublished data 2004) were therefore analysed to generate locally appropriate age-based dosage guidelines chosen to minimize under- and over-dosing while keeping simple age categories, and avoiding the use of quarter tablets. Breakable, non-dispersible tablets of AQ (200 mg) and SP (500 mg sulphadoxine/25 mg pyrimethamine) were used. The analysis indicated that children aged 2 to 11 months should receive half tablets of SP and AQ on the first day, followed by half tablets of AQ on the two subsequent days, and that from age 1 to 4 years, children should receive whole tablets. These findings conflicted, however, with the nurses’ experience, as when this drug combination was Senegal’s interim first-line treatment, the national guidelines indicated that whole tablets should only be given from the age of two years. Given local health workers’ concerns about side effects of SP and despite researchers’ concerns about under-dosing, agreement was reached that whole tablets would only be given from 2 years, and that care would be taken to ensure that only children under 5 would be included in the campaign, as older children, if treated with the dose for 1–4-year-olds, could be under-dosed (Table S1).

Supervision of all three daily doses was considered impractical and the health workers highlighted that mothers were already experienced in administering curative multi-day drug regimens, including antibiotics and antimalarials, at home. The consultative group therefore decided that CHWs should demonstrate for the child’s caregiver proper administration of the first day’s tablets, and that the caregiver could administer the two remaining daily doses of AQ unsupervised at home. The breakable, non-dispersible tablets were to be crushed and mixed with sugar and water using plastic cups, spoons, and sugar carried by the CHWs and the household’s usual water supply.

AQ was known to be associated with an increased risk of vomiting, and because of AQ’s bitter taste, some of the children were likely to spit out some of the medicine. Children were re-dosed by the health worker if the first dose was vomited while the health worker was present. However, given that it would often not be clear how much of the medicine had been ingested, caregivers would be advised not to re-dose, to avoid the possibility of overdosing, and avoiding the complication of providing families with repeat doses.

**Training community health workers:** Members of the district health management team, the research team, and head nurses collaborated to lead separate, one-day trainings for CHWs in each of the three pilot health posts. In addition to eligibility criteria, dose, and drug administration, the CHWs were also trained in communication, side effects, record keeping, and operational issues.

**Communication with local communities:** Each health post nurse, in agreement with their health committee, organized a meeting with village heads and local leaders to explain the activities and timing of monthly rounds. Each village head was asked to circulate information within their village. Additional social mobilisation activities were organised in each health post area. In one health post, communities were informed through church services and in mosques, and a caravan toured the villages - village criers (*griots*, *criers*) hired for 5 days to circulate on horse carts in the intervention villages with loudspeakers to deliver (often witty!) slogans regarding the upcoming SMC delivery. In the other health posts, local meetings were held in different parts of the health post area by the nurse and by CHWs several days before the start of SMC delivery.

During SMC delivery, CHWs explained the strategy, asked for consent, and explained how to administer treatment doses. CHWs were trained to make caregivers aware of symptoms of known side effects and to advise them to bring the child to the clinic immediately if she/he was unwell after SMC treatment. CHWs were also trained to explain that no malaria interventions are completely effective, that all members of the household should sleep under a treated bed net, and that if the child is unwell with a fever, it could be malaria, so health care should be sought immediately. CHWs were trained to ask before giving drugs about history of allergies to medicines and side effects to previous intake of SMC drugs.

**Record keeping:** The CHWs distributed SMC cards to all families in September 2006. When these were not retained in subsequent years, CHWs began noting receipt of SMC on children’s vaccination cards instead. Tally sheets were used by each CHW pair to record the number of each type of tablet received, used, and returned, and numbers of treatments administered by age group. Village registers were used to provide a record of all the monthly treatments a child received. Supervision sheets were used to summarise the number of children treated by age group, and tablets used and returned, by each CHW team.

**Logistics:** CHWs themselves highlighted the importance of working in pairs, with one explaining the intervention to the family and administering the drugs, and the other checking the child’s eligibility and completing the family’s record card and the administrative tally sheet.

The health posts nurses were responsible for working out each CHW pair’s area of responsibility, route, and target number of children to be reached each day, as they were experienced in this type of planning for other campaigns.

Each CHW pair took bags of tablets, a bag of sugar, 2 cups, 2 spoons for crushing the tablets, a register, and forms for recording drug usage. They were also told to return to the household if a child was declared absent. During visits the following month, they were asked to check for adverse events and document vomiting, refusals and absence on a register.

The CHWs were paid at daily rates similar to other campaigns, 3500 CFA per day (=US$ 7.5 in 2006), for 3 days each month over 3 months. This rate, which included transport costs, was proposed by the district medical officer and agreed with the CHW union.

**Supervision and monitoring:** The district public health officer, district medical officer, or the research team’s district supervisor visited each health post once per day during SMC administration and PIs (BC, PM) visited monthly.

#### Pilot study data collection and analysis

In addition to the routine monitoring data, a cross-sectional survey was conducted at the end of the transmission season to gather data on receipt of SMC, access to health care, use of bed nets, socio-economic status, vaccination history, and nutritional status. A total of 686 children living in the catchment areas of the 4 health posts were selected for inclusion using a form of probability sampling that did not require a sampling frame: 40 clusters representing 32 villages in the pilot area were selected with probability proportional to the number of children under 5 estimated in district health records. Rough sketch maps were then used to divide the 40 clusters into geographical segments of approximately equal numbers of dwellings, from which 40 segments were selected by simple random sampling and all children under five in each of the 40 segments were included.

To monitor for adverse events and check compliance with the unsupervised AQ doses on days 2 and 3, fieldworkers visited a systematic sample of children aged 2 to 59 months (as of September) on day 4 in November (i.e. three days after the SMC distribution). The sample was stratified by health post and caregivers of 636 children in the 3 implementing health posts and of 216 children in the control post were interviewed. In addition, passive surveillance in health facilities was also used to monitor adverse events, which are discussed in greater detail elsewhere^8^.

Malaria cases were detected through passive surveillance using rapid diagnostic tests (RDTs) in the participating health posts.

A series of interviews and focus group discussions were used to explore the acceptability of the intervention to health workers and communities. Direct observation was done in homes to observe interactions between health workers and caregivers and the administration of supervised and unsupervised SMC doses.

#### Ethics

The pilot study and main trial were approved by the ethics committee of the London School of Hygiene & Tropical Medicine and by the Conseil Nationale de Recherche in Senegal. Before the pilot study, and for the main study, meetings were held with local authorities and health staff to explain the aims and activities of the research. On the first occasion, when the intervention drugs were delivered through house-to-house visits, verbal consent to participate was sought from the mother or carer of each eligible child using a standard script translated into the appropriate local language which explained the purpose of the project, the purpose of the drug treatment, the common side effects that might be experienced and that severe side effects, while possible, are unlikely. Consent was documented in a register by the community worker. Consent was sought separately for participation in surveys after explaining the procedures involved using a standard script. The studies followed good clinical practice guidelines.

### Pilot study results

In total, children received 5059, 5209, and 5292 courses of SMC in September, October, and November 2006, respectively, and 4635 children received all three intended courses of treatment. An estimated 81% (95% confidence interval: 76% to 85%) of eligible children received all 3 scheduled courses of treatment, while 88% received at least one dose (Table 2).

**Table 2:** Pilot study: coverage in Tivaouane District, 2006.

Coverage (95% CI)	Health post			
	Cherif Lo	Medine	Pambal	Total
Sep	86%	72%	91%	85% (80–90%)
Oct	91%	74%	93%	89% (84–93%)
Nov	89%	75%	92%	87% (84–91%)
All 3 monthly courses	81%	67%	86%	81% (76–85%)

**Figure 2:**
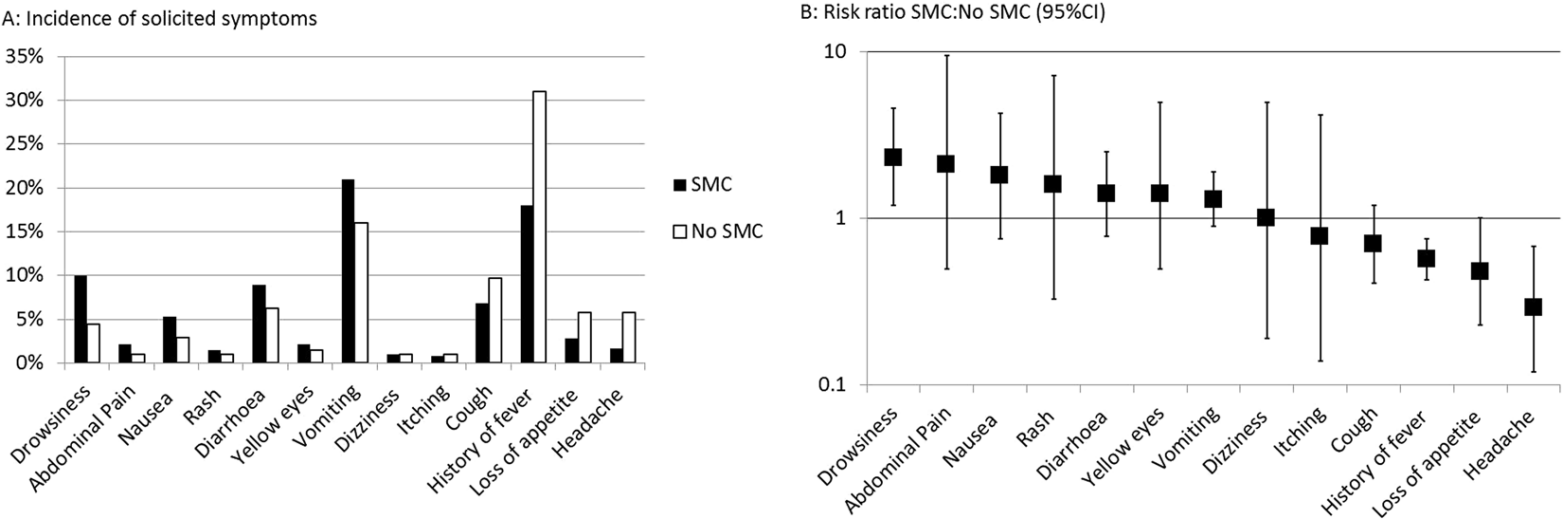
(**A**) Percentage of children in whom caregivers reported symptoms, 4 days after SMC distribution, in children who received SMC and in children in control areas who did not receive SMC, (**B**) Risk ratio for each reported symptom (SMC:No SMC);ratios above 1 indicate increased risk in children who received SMC).

Amongst children in the 72 intervention villages (population < 5yrs approximately 5630), 4 RDT-confirmed cases of malaria were detected, as compared with 25 confirmed cases in the control villages (population 1153), giving an incidence rate ratio of 0.03 (95%CI 0.015,0.07). Incidence of solicited adverse events recorded on day 4 after the initial dose of SMC (Fig. 2) was similar amongst children who received SMC and children who did not for all symptoms (including vomiting), except for drowsiness, which was more common in children who received SMC (risk ratio 2.3, 95% CI 1.2–4.6), and caregiver-reported fever and headache which were both less common in children who received SMC (risk ratio for fever: 0.57, 95% CI 0.43 to 0.76, and for headache 0.29, 95% CI 0.12 to 0.68).

The most common reason for not receiving SMC was being away from the village at the time of delivery. Amongst children who missed one or more courses of treatment, 56% of children and/or their mothers were temporarily away from home or not living in the village at the time of delivery; in only 4% of cases did parents refuse and in 6.5% of cases the mother reported that the CHW did not visit her house. Of the 513 mothers interviewed on day 4 whose child had received the supervised dose on day 1, 5% said they had not given the day 2 dose of AQ and 10% said they had not given the day 3 dose; of these, half said they had forgotten, while 11% attributed the omission to the bad taste, 10% to the mother’s absence, 9% to vomiting, and only 2% to the child’s refusal. 80% of mothers had retained the blister pack for inspection, of these, 12% had left-over medication. Drawing on the qualitative research, the team identified several ways in which implementation could be improved, which are discussed in the next section.

During direct observation, supervision of the first dose was generally adhered to, but one instance was observed where the *relais* did not supervise the first dose, leaving the drugs with the caregiver. Some mothers with children of different age groups found it difficult correctly to distinguish the dose for each child. Community sensitization did not reach some households and the measures employed in some health post areas (where the mobile caravan was used) were more effective than those relying solely on local meetings, although the latter were important in allowing the opportunity to answer questions from the community. Explaining the nature of the common side effects and that these were generally mild and short-lived, was important in promoting adherence and to avoid spread of rumours. In general, the intervention was well accepted. Malaria was felt to be the major health concern, and parents were particularly concerned for their children’s health and recognized that bednets provide only partial protection, and therefore welcomed the use of preventive medicines. Despite the bitter taste of AQ, high coverage was achieved with good adherence to both supervised and unsupervised doses.

Initial costing at the pilot stage indicated costs of delivery were likely to be affordable.

Findings were presented at a meeting of the project team at which the Director of Medical Services, the regional medical officer, and the research focal point of the national malaria control programme, and representative of the funder were present. Key messages from the study were fed back to participants in a community meeting. The Director of Medical Services then chaired a working group including heads of the departments of community health and of nutrition, district medical officers from the study areas, and members of the research team (PM, BC), which considered delivery approaches for a potential national scale-up, and whether SMC could be delivered alongside other interventions such as Vitamin A and deworming.

From 2007 to 2009, SMC was delivered in all 4 health posts in the pilot area, in 2010 SMC was discontinued in these health posts and implementation started in four other health posts in the same district with a higher burden of malaria.

The intervention had been known as Intermittent Preventive Treatment in children (IPTc), or *Traitement Préventif Intermittent* (TPI) *chez les enfants*. From 2008, in Senegal it was referred to as PSP (*Prévention Saisonniare du Paludisme*) to avoid any confusion with IPT in infants which the country had decided not to adopt.

## Large-Scale Implementation

### Large-scale implementation methods

The design of the stepped wedge trial is described in Cisse *et al.*^5^ and NDiaye *et al*.^8^. Briefly, 54 health posts were randomized with respect to the timing of when SMC would be introduced, 9 to start in 2008, 18 to start in 2009, 18 in 2010. The remaining 9 did not implement SMC.

#### Large-scale delivery

Implementation methods built on the pilot study with the modifications described below. These adaptations addressed findings from the pilot study, changes in epidemiology, difficulties as they arose, and the challenges of gradually scaling up delivery while scaling back research team support over three years, so that in 2010, district health management teams could deliver SMC largely independently in 46 health posts. Early in 2010, members of the research team attended a meeting with the Global Malaria Programme of WHO, at which available evidence on IPTc was discussed preparatory to convening a review by the WHO Technical Expert Group on chemoprevention. A key point was the need to ensure that use of antimalarial drugs for preventive treatment did not undermine the policy that use of antimalarial drugs for case management should be based on a positive parasitological diagnosis (rather than presuming fever indicated malaria). For this reason, in the 2010 implementation, there was added emphasis on the need to check if the child had a fever, and refer all febrile children so that they could be tested for malaria at the health facility and then treated with ACT if positive, or given SMC treatment if they were negative.

**Communication:** A series of meetings were held with the local government authorities and district health staff to explain the aims and activities of the project. In each of the three districts of the project area, the *préfet* (the senior local government administrative officer of the *département*) convened meetings of the presidents of rural communities and *sou-préfets* in his or her *département*, at which the aims and activities of the project were explained using an information sheet.

Each medical district developed its own communication plan and money was given to be used for raising community awareness about the intervention. As the importance of informing caregivers of the timing of SMC days before every round was a lesson learnt from pilot study, activities were organised before the first round in September and again before each of the subsequent courses. In Fatick, Niakhar and Mbour, information about the intervention was broadcast on local radio stations, and phone-in programmes were organised in which project staff answered questions. In Bambey, *griots* (village criers) went to selected villages accompanied by the district public health officer. In all districts, village meetings were organised, heads of neighbouring villages were invited, and question and answer sessions were led by the district public health officer, the district medical officer or members of the research team. As in the pilot study, the importance of providing opportunities for questions to be asked was a key finding. These activities were facilitated by the *Préfets* and *sous-Préfets* and the *Présidents des Communautés Rurale* who provided some of the transport and logistics for these activities. To improve awareness about the intervention and in response to qualitative findings in the pilot, CHWs were given T-shirts and caps bearing project insignia, which they wore during the SMC rounds.

**Eligibility:** From 2008, the lower age limit was raised from 2 months to 3 months because of the low risk of malaria in very young infants. From 2009, in response to the changing malaria epidemiology which was then apparent, the upper age limit was extended from 59 months to 119 months.

**Adapting the delivery approach to include older children:** A further consultation was held between district medical officers, health post nurses, community health workers and researchers to discuss implications of extending the programme to include older, school-age children. Delivery through schools was considered, this would be relevant only for the last month of delivery as other months are outside school terms, but was not pursued as it would exclude children not enrolled and those not attending school when SMC was delivered. Delivery would be scheduled at times when older children were likely to be at home, on Fridays, evenings and weekends.

**Drug dosage and formulation:** With the expanded age range from 2009, a third age category was created for dosing. Children aged 72 to 119 months, should receive 1.5 tablets of AQ and SP on day 1 and 1.5 AQ tablets on days 2 and 3. Also from 2009, the composition of SP was changed from sulfalene to sulphadoxine because of a change in supplier. In October and November 2010, a different formulation of SP and AQ was used which was not only breakable, but also dispersible and sweetened, to facilitate administration (Table S1).

**Drug administration:** In 2008, the CHWs were provided with cups and spoons with which to administer the tablets as they had been in the pilot study, however, this raised concerns amongst carers about hygiene, and also seemed an unnecessary expense for the programme. From 2009, CHWs therefore used the household’s own cups and spoons, and provided the tablets to older children to be swallowed whole under observation.

**Training head nurses and community health workers:** In 2008, all 9 head nurses whose health posts were randomized to implement SMC gathered in the IRD research center of Mbour for a 2-day training. BC and EHB led the training, while the four head nurses who implemented the pilot were also present and shared their experiences. In 2009 and 2010, head nurses who implemented SMC in 2008 trained those who were implementing SMC for the first time.

In all three years, each implementing health post held a one-day training session on the day preceding the commencement of the September round of SMC delivery.

**Record keeping:** Registers were used to record monthly treatment, and tally sheets to record number of treatment doses. With the implementation of a demographic surveillance system (DSS, described below) from 2008, families were issued with SMC record cards, one for each caregiver, on which SMC treatments of children in their care were recorded.

**Logistics and timing:** Administration was planned to begin on the same date in all health posts each month and year. The 5-day period at the middle of each month was chosen taking into account public holidays, and other health activities of district staff. Community sensitization and local mobilisation were organised by the district communication officer. In September 2010, each health post was provided with a printed register for each village in their catchment area, listing eligible children.

**Drug procurement and management:** In 2008, drugs for SMC (Dualkin, sulfalene-pyrimethamine and amodiaquine) were donated by Pfizer, Dakar; sulfalene is chemically very similar to sulfadoxine. Drugs were purchased in 2009 from planet Pharma, Paris representing Chongqing Qinyang Pharmaceutical Co Ltd and in 2010 from Kinapharm (AQ 153 mg and SP 500 mg) and National pharmacy of Senegal (SP 500 mg).

A sample of SP and AQ tablets was tested in Dakar (*Laboratoire des pharmacies et des médicaments*) and London (London School of Hygiene & Tropical Medicine) and passed standard criteria for drug content, uniformity of content, dissolution and impurities.

Drugs were stored in the research facility in Mbour under supervision of the field research coordinator. The quantity of drugs needed for each district was calculated based on the population of children in each age-based dosing group plus a margin of 20%. Populaton estimates came from a census done in 2008 updated through demographic surveillance rounds. District staff or, in some cases, the health post nurses transported the drugs to the health posts at least 3 days before drug delivery was due to commence each month. The day before drug delivery each month, CHWs at each health post placed courses of tablets appropriate for each age group into plastic bags. Remaining drugs from each round were kept in health posts after the September and October campaigns and the additional tablets required given before the following month based on number of children seen during the previous month. At the end of the November rounds, all remaining drugs were sent back and stored in a controlled temperature room at the research health facility in Mbour.

**Supervision and monitoring:** At end of each day, the CHWs reported to the head nurse the number of children seen and drugs remaining, and checked the register for any children who had been missed. The nurse then tallied the total number of AQ and SP tablets administered, counted the number of tablets remaining, calculated wastage, and, if less than the predicted number of tablets had been used, sought reasons for the discrepancy (e.g. refusals, errors in listings, migration of families). The nurse then reported this information to the district medical officer each day by mobile telephone and issued CHWs with drugs for the next day.

In 2008, the 9 implementing health posts continued to receive daily supervisory visits during the delivery days from either district or research staff. In addition, project DSS (Demographic Surveillance System) interviewers visited each nurse and each CHW team in their DSS circuit once per SMC round to check the team had forms and registers and used them correctly, had enough drugs, sugar, and other supplies, and were correctly dosing by age. In 2009 and particularly in 2010, however, as the number of health posts increased, the research team scaled back their support such that the DHMTs supervised largely unaided.

**Pharmacovigilance:** A series of steps were taken to strengthen the national PV system in the study districts^8^.

**Costing:** A detailed costing study was undertaken in 2010, reported elsewhere^9^.

#### Data collection

Data sources comprised a demographic surveillance system (DSS), administrative data, a series of cross-sectional household surveys, and a survey of CHWs. Data were recorded on paper forms and scanned into databases using the Teleform system.

**Demographic Surveillance System:** A census of the study area was carried out in March-May 2008. After data entry and cleaning, all households were revisited in August to give each mother or caregiver a card bearing an identification number for the household, for the mother/caregiver, and for each child in her care, with space to record information on the administration of SMC, Vitamin A, and mebendazole. Health facilities were provided with blank DSS cards to issue to first-time mothers at their first contact with health staff after delivery, and annual rounds of household visits were conducted to record changes in household occupancy.

**Administrative data:** Tools to document the drug administration process (quantity of drug given, date and age group) were developed and given to each team of CHWs. Register were printed out from the demographic surveillance system data base with information to identify each child (mother’s name, name of head of household, household number, village name and number - with columns to document presence, absence, refusal of treatment, and whether the child vomitted). The registers were printed once per year with columns on each page to collect information for each child each month. Tallies were made from registers to determine the number of refusals, and absent and sick children each round each year.

**Cross-sectional household surveys:** Surveys were conducted at the end of the malaria transmission season in December 2008, 2009, and 2010 to determine coverage of SMC and reasons for missed doses, adherence to daily doses in the most recent SMC treatment, to record bed net use by children after inspecting the place where the child slept, to measure the prevalence of parasitaemia and anaemia, and to ask about any adverse events related to SMC. In 2008, 5 villages in the catchment area of each of the 54 health posts implementing SMC were selected with probability proportional to size. In each selected village, households were selected by simple random sampling from the Demographic Surveillance System (DSS) database to yield at least 20 children in each village, giving a minimum sample of 100 children per health post, or 5,400 in total. In 2009, the sampling strategy was modified somewhat: households were selected in each health post’s catchment area by simple random sampling from the DSS database to give a sample of at least 130 children per health post, resulting in an actual total sample size of 6,827 children. In 2010, to improve the power for analysing parasitaemia prevalence, the sampling strategy was again modified to over-sample the 9 remaining control health posts and those with higher malaria incidence by weighting each by a factor of 2 before randomly selecting 40 health posts with probability proportional to size. In each selected health post, households were selected by simple random sampling to yield about 25 children in each health post and 1000 children in total.

The DSS database was used to determine the number of households to sample in each village or health post to generate the required sample size. A list of all eligible children in the selected households was prepared from the DSS database, but interviewers were instructed to recruit all children within the appropriate age range normally resident in the household, regardless of whether they were in the DSS listing or had received SMC. “Normally resident” was defined as children who had been resident for at least 6 months or, if resident for less than 6 months, planned to remain in the area for the next 6 months. In December 2008, all normally resident children aged (when surveyed) 6–63 months in the sampled households were included in the survey, and in December 2009 and 2010, all normally resident children aged (when surveyed) 6 to 123 months were included. While children who reached the age of 3 months after the September SMC round, but before the October or November rounds were eligible to receive SMC in the later months, they were not included in the December survey. In all surveys, the sampling fraction varied between health posts in order to yield an approximately constant number of children per health post. If the target number of children was not reached after all the selected households had been visited and call-backs completed, an additional listing of households, selected by simple random sampling from the same health post, was used. Sampling continued until the target sample size was reached.

The survey was conducted over 14 days each year. Interviewers alternated between health posts that had received SMC and those that had not to avoid systematic bias in the timing of the interviews. One call-back visit was arranged if the mother or a child was absent.

The mother (or other caregiver) of each child was asked about her demographic characteristics, education, sources of income, household and personal assets, type of house and availability of electricity, telephone and water supply. For each child, the duration of residence in the village was recorded, and the place where the child slept the previous night was inspected to record the type and condition of the net. In areas where SMC had been delivered, the number of SMC courses received was recorded from the DSS card if available or from mother’s recall. If scheduled SMC doses were not received, the mother was asked the reasons.

**Community health worker survey:** At the end of 2010, a survey was conducted of a sample of 47 CHWs, from all four districts, addressing their experience of SMC delivery. They were asked what questions were most commonly raised by caregivers; what they did if the child was febrile; whether they enquired, before administering SMC, if they child had had adverse reactions to SMC drugs previously or had known allergy to SMC drugs; whether cups, spoons, water and sugar were provided by the household or the health post; the steps taken to treat school-age children during term time; how they determined the child’s age group for dosing (under 2 yrs, 2–5 yrs, 6 yrs and over) when the child was not in the DSS listing; whether they had knowingly treated children older than 10 years; what happened if the mother said there was a child that should be treated but the child was not at home when the CHW came; whether they consistently checked if the child had received other medicines in the last month, specifically SP or AQ or bactrim, and whether or not they administered SMC in such cases; and if the child was currently on a course of antimalarial treatment, whether they administered SMC. It was explained that the aim of the survey was to determine what was done in practice and there were no right or wrong answers.

#### Data management and analysis

**Coverage levels:** Administrative data on the number of courses administered was tallied from health centre registers and aggregated by district. Reports from health posts and delivery tools developed to document day to day activity of drug delivery teams were collected and compared with registers to check for consistency.

Survey questionnaires were checked for completeness by the district supervisor before being batched and sent to Dakar for scanning using the Teleform system and export to Access databases. The proportion of eligible children receiving 0, 1, 2, and 3 courses of SMC and 95% confidence intervals were estimated from the survey data using a ratio estimator, with each observation weighted by the inverse of the sampling fraction for the health post using Stata version 11 (Statacorp, College Station, Texas). For 2009 and 2010 data, coverage levels were also estimated by age group to compare annual coverage for children aged 3 to 59 months with coverage of those aged 60 to 119 months in September of each year; p-values were calculated using the designed-based F test.

**Equity:** Equity of coverage was conceptualized as children having the same probability of receiving SMC regardless of their age (within the target age range), gender, the household’s socio-economic status (SES), or their mother’s education type or level. Analyses were based on the cross-sectional household surveys. Each child’s SES quintile was assessed using principal components analysis of his or her mother’s assets (ownership of cattle, sheep, goats, horses, donkeys, radio, television, video cassette recorder, watch, telephone, metal or wooden bed, bicycle, cart, motorbike, car), household construction (roof material, floor material), and household amenities (running water, electricity, fixed phone line, flush toilet, pit latrine, solar power, cooking fuel). The proportion of children who had received all three intended courses of SMC and the proportion who had slept under a bed net (a long-lasting insecticide-treated bed net (LLIN), any treated or impregnated bed net, or any net) the previous night were then disaggregated by SES quintile. Logistic regression was used to calculate the change in odds of coverage for a one quintile increase in SES. Test for trend for SES was done using logistic regression model fitting SES as a linear effect and accounting for survey design using STATA survey commands. Coverage of SMC was also compared across the level and type (none, Koranic, or French) of the mother’s education. Differences between groups were compared using design-based p-values.

#### Ethics

The trial protocol was approved by Senegal’s Conseil National pour la Recherche en Santé and the ethics committee of the London School of Hygiene & Tropical Medicine. The trial is registered at www.clinicaltrials.gov, number NCT 00712374.

During each round of SMC, CHWs explained the aims of the project, the purpose and potential side effects of the study drugs, using a standard script translated into the appropriate local language (Wolof or Serer), sought verbal consent from the mother or caregiver of each child, and recorded consent or refusal in a register.

Consent was sought separately for participation in cross sectional surveys. After publicizing the survey in the community through meetings with CHWs, the village head, and public meetings, DSS interviewers visited selected households. They explained the aims and procedures of the survey, using a standard information sheet, and sought written consent from the mother or caregiver of each eligible child.

### Large-scale implementation results

#### Courses of SMC delivered

In the three years of the main implementation study, children aged at least 3 months and under 10 years received more than 780,000 courses of SMC. (Table 3) The number of courses received reflects instances in which a CHW successfully administered or observed a child take the first day’s dose of AQ and SP and provided AQ tablets for administration on the two following days. In a relatively small number of additional instances, a child was reached by a CHW, but the parents or child refused (7,660, 0.97% of contacts), the child vomited or spat out the first dose (892, 0.11%), or the child was unwell and therefore ineligible to receive SMC, in which case the child was referred to a health facility (314, 0.04%). In addition, 7% of targeted children were absent in 2008 and this rose to 13% in 2009, but then dropped to just 3% in 2010 (Table 3).

**Table 3:** Effective coverage: Receipt of SMC and reasons for not receiving.

Month	2008 (children 3–59 months, 3 health posts)				2009 (children 3–119 months, 27 health posts)				2010 (children 3–119 months, 46 health posts)				TOTAL
	Sep	Oct	Nov	Total	Sep	Oct	Nov	Total	Sep	Oct	Nov	Total	
Received SMC	16,218 (90.31%)	15,756 (91.77%)	14,764 (90.62%)	46,738 (90.89%)	86,949 (85.32%)	86,514 (84.88%)	88,553 (85.04%)	262,016 (85.08%)	154,014 (96.57%)	157,602 (96.63%)	159,667 (96.63%)	471,283 (96.61%)	780,037 (92.07%)
Absent	1,544 (8.60%)	1,114 (6.49%)	1,079 (6.62%)	3,737 (7.27%)	14,147 (13.88%)	13,956 (13.69%)	13,079 (12.56%)	41,182 (13.37%)	4,600 (2.88%)	4,411 (2.70%)	4,385 (2.65%)	13,396 (2.75%)	58,315 (6.88%)
Refused	45 (0.25%)	145 (0.84%)	292 (1.79%)	482 (0.94%)	649 (0.64%)	1,317 (1.29%)	2,429 (2.33%)	4,395 (1.43%)	655 (0.41%)	1,007 (0.62%)	1,121 (0.68%)	2,783 (0.57%)	7,660 (0.90%)
Vomited or spat out	142 (0.79%)	146 (0.85%)	145 (0.89%)	433 (0.84%)	68 (0.07%)	55 (0.05%)	35 (0.03%)	158 (0.05%)	177 (0.11%)	72 (0.04%)	52 (0.03%)	301 (0.06%)	892 (0.11%)
Unwell	10 (0.06%)	8 (0.05%)	13 (0.08%)	31 (0.06%)	94 (0.09%)	82 (0.08%)	38 (0.04%)	214 (0.07%)	41 (0.03%)	13 (0.01%)	15 (0.01%)	69 (0.01%)	314 (0.04%)
TOTAL	17,959 (100.00%)	17,169 (100.00%)	16,293 (100.00%)	51,421 (100.00%)	101,907 (100.00%)	101,924 (100.00%)	104,134 (100.00%)	307,965 (100.00%)	159,487 (100.00%)	163,105 (100.00%)	165,240 (100.00%)	487,832 (100.00%)	847,218 (100.00%)

Findings are presented from administrative data.

#### Quality of delivery

At each health post, SMC delivery took between 2 and 6 days each month and involved between 4 and 68 CHWs. Each pair of CHWs administered SMC to approximately 54 children per day in 2008 (Table 1). As virtually doubling the age range only increased the number of households to visit by 13%, the average number of children receiving SMC from each pair of CHWs increased to 78 per day in 2009 and 88 per day in 2010.

The system of daily supervisory visits at every health post on every administration day in 2008 picked up a number of errors promptly. For example, in September 2008, an excess of SP tablets had been used in 3 health posts, and the supervisor discovered that this was because SP as well as AQ had been left with the mother for administration at home on days 2 and 3. In response, CHWs were sent back the same evening to collect the SP, all nurses were telephoned to remind them of procedures, and training in future years further emphasized the dosage, such that the problem did not recur.

Staff turnover was low both amongst head nurses and CHWs. Only 5 of 46 nurses left their health post during the three years, and three of these 5 remained in the study area.

In the resource use and activity survey conducted in 2010, nearly all CHWs declared that this was their first time (from 2008) delivering SMC. Questions most commonly asked by caregivers were about the purpose of SMC; why adults were not included; why these drugs (SP, AQ) were used; why children can become unwell after taking the drugs and questions related to side effects; and whether SMC would eliminate malaria. 37/47 (79%) of CHWs said they consistently referred febrile children to the health post and did not give SMC treatment, but 4 (9%) had treated febrile children and 7 (15%) had with-held treatment without referring. Checking for previous side effects or known allergies to SMC drugs was not routinely done. It was stated by several CHWs that this was because they felt that if there had been side effects, mothers would volunteer this information without being asked. 38 (81%) said that the sugar they used in the previous SMC was provided by the project and 9 (19%) said they used sugar provided by the household. When asked about steps they had taken to treat school-age children, 36 (77%) said they had returned to the household in the afternoon, 4 (9%) said they had made appointments for the following day, 7 (15%) said treatments had been given at school supervised by the school director, and 4 (9%) said they had left drugs for this child with parents. For determining the dose to use, vaccination cards were checked but if these were not available, the age was estimated by comparison with other children in the compound. One CHW (2%) had treated children above 10 years of age. To treat children who were not present at the time of the visit, 27 (57%) mentioned making an appointment to return the following day, 13 (28%) mentioned leaving the treatment with the mother, and 7 (15%) mentioned going to find the child nearby. None of the CHWs surveyed said that they checked if the child had received SP, AQ or Bactrim in the previous month. Most (89%) said they would not administer SMC if the child was currently on a course of antimalarial treatment, but 5 (11%) said they would.

#### Levels of coverage

According to administrative data and DSS population estimates, average monthly coverage increased from 76% in 2008, to 83% in 2009, and up to 87% in 2010 (Table 4). Coverage varied from a low of 65% of children in the target age range (3 to 59 months) receiving SMC in Mbour district in November 2008 to a high of 92% of children in the target age range (3 to 119 months) receiving SMC in Bambey district in November 2010.

**Table 4:** Effective coverage by district.

District	Year	2008			2009			2010		
	Month	Sep	Oct	Nov	Sep	Oct	Nov	Sep	Oct	Nov
	Number of health posts implementing	9			27			46		
	Target age range	3–59 months			3–119 months			3–119 months		
Bambey	Courses of SMC administered	4,677	4,250	4,104	38,673	38,583	41,102	74,047	76,018	77,292
	Target population	5,092	5,092	5,092	46,208	46,208	46,208	83,756	83,756	83,756
	Coverage	92%	83%	81%	84%	83%	89%	88%	91%	92%
Fatick and Niakhar	Courses of SMC administered	5,189	4,903	4,912	27,253	26,908	26,182	45,565	46,274	47,375
	Target population	6,482	6,482	6,482	32,659	32,659	32,659	53,863	53,863	53,863
	Coverage	80%	76%	76%	83%	82%	80%	85%	86%	88%
Mbour	Courses of SMC administered	6,352	6,603	5,748	21,023	21,023	21,269	34,402	35,310	35,000
	Target population	8,836	8,836	8,836	26,507	26,507	26,507	43,441	43,441	43,441
	Coverage	72%	75%	65%	79%	79%	80%	79%	81%	81%
Total	Courses of SMC administered	16,218	15,756	14,764	86,949	86,514	88,553	154,014	157,602	159,667
	Target population	20,410	20,410	20,410	105,374	105,374	105,374	181,060	181,060	181,060
	Coverage	79%	77%	72%	83%	82%	84%	85%	87%	88%

Coverage estimates are based on administrative data and DSS population estimates.

Cross-sectional surveys indicated that in 2008, 92% (95% Confidence Interval: 89%, 95.5%) of children in the target age range (3 to 59 months) received all three monthly courses of SMC (Table 5). In 2009, when both the target area and age range increased, estimated coverage decreased to 84% (82%, 87%) of targeted children receiving all three courses of SMC. In 2010, estimated coverage with all three monthly courses of SMC increased to 93% (91%, 96%) of children in the targeted age range and areas, the highest level achieved in any of the 5 years of the study. Fewer than 5% of children received one or two courses of SMC in any given year. The proportion of children captured in the end-of-season surveys who were reported not to have received any courses of SMC increased from 3.5% (2%, 5%) in 2008 up to 11% (9%, 13%) in 2009, and then decreased to 6% (4%, 8.5%) in 2010. Estimated coverage in each month is shown in Table S4.

**Table 5:** Effective coverage by age group.

Age range	Year	2008 (N = 1,019)		2009 (N = 3,397)		2010 (N = 882)	
	Number of courses received	Point estimate	95% CI	Point estimate	95% CI	Point estimate	95% CI
3–59 months	0	3.5	1.9, 5.1	12.9	10.4, 15.5	8.8	4.9, 12.8
	1	0.9	0.2, 1.5	2.2	1.4, 3.1	0.4	−0.2, 1
	2	3.3	0.8, 5.8	3.3	2.0, 4.6	0.4	−0.1, 0.9
	3	92.3	89.1, 95.5	81.6	78.4, 84.8	90.4	86.4, 94.3
60–119 months	0	NA	NA	9.4	7.3, 11.4	3	1.3, 4.6
	1	NA	NA	0.9	0.3, 1.4	0	0, 0
	2	NA	NA	2.7	1.5, 4.0	0.7	−0.1, 1.6
	3	NA	NA	87	84.5, 89.6	96.3	94.4, 98.2
Overall coverage	0	3.5	1.9, 5.1	11.1	9.2, 13.0	6.1	3.7, 8.5
	1	0.9	0.2, 1.5	1.5	1.0, 2.0	0.2	−0.1, 0.5
	2	3.3	0.8, 5.8	3	1.8, 4.1	0.6	0.1, 1.0
	3	92.3	89.1, 95.5	84.4	81.9, 87.0	93.1	90.6, 95.6

Coverage estimates are based on end-of-season cross-sectional survey data. In 2008 SMC was only given to children under 5 years. Children missing information on the number of SMC courses received are assumed to have received no courses. Number of courses received was not missing for any children in 2008 or 2010. Number of courses was missing for 5 children (0.15%) in 2009. *Age was missing for two children in 2009 and one child in 2010, and so these three children are reflected in overall coverage, but not the age-specific coverage estimates. Design-based test of homogeneity of proportions by age group: 2009, p < 0.001; 2010, p = 0.0018.

For all three years, the cross-sectional survey data provided higher coverage estimates than the combination of our administrative data and DSS, which likely reflects differences in denominators. The DSS estimated the total population normally resident in the study area, whereas the cross-sectional surveys excluded people who were temporarily absent at the time of the surveys. The DSS estimates are therefore likely to have overestimated the target populations and underestimated coverage, because some families had temporarily migrated, and were therefore not present to receive SMC and potentially not even exposed to malaria.

The surveys also indicated a notable dip in coverage in 2009, whereas the administrative data indicated continuous increases in average yearly coverage. This discrepancy likely relates to the relatively high proportion of children who were absent during SMC delivery in 2009 (15%) as compared with 2008 (8%) or 2009 (3%) (Table 3); perhaps because of less effective communication, families may have been temporarily away from home on the delivery days in 2009, but nonetheless captured in end-of-season survey that year.

In 2009, 9.6% of caregivers reported difficulty administering the unsupervised doses; this percentage was slightly lower for children 5–9 years (7.4%) than for under-5’s (12%). The most common problems (76% of those reporting difficulties) were refusal or rejection by the child. Other reasons included the caregiver being away, and the child being unwell. Only 2% said they had forgotten to give the two doses or had lost the tablets.

#### Equity of coverage

In 2009, coverage was somewhat higher amongst children aged 5 to 9 years (87%, 95% confidence interval 84.5%, 90%) than amongst children under 5 (82%, 95%CI 78%, 85%, p < 0.001). Although coverage increased substantially in both age groups the following year, coverage remained higher in older children (p = 0.002, Table 5).

**Figure 3:**
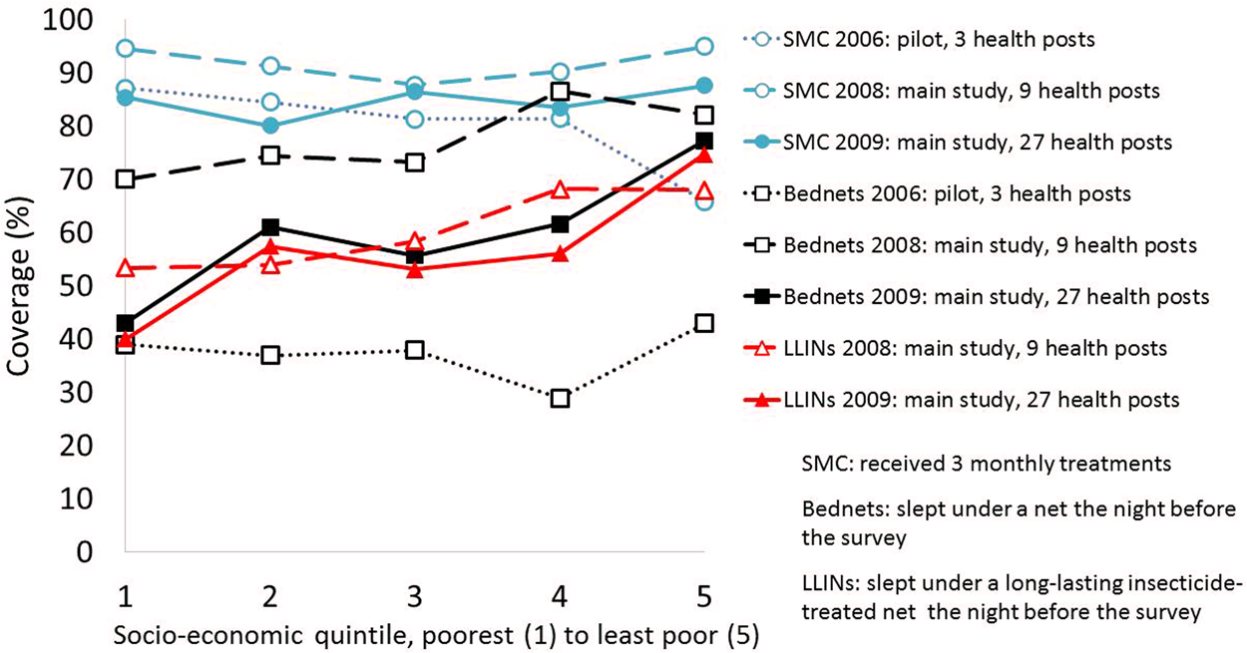
Equity of receipt of SMC and use of bed nets by socio-economic status in 2008 and 2009 (Under-5 s in 2006 and 2008, under-10 s in 2009. LLIN: Long-lasting insecticide-treated bednet).

Figure 3 shows that there was no evidence of a linear trend in the probability of receiving all 3 courses of SMC across SES quintiles in either 2008 (p = 0.63) or in 2009 (p = 0.36), the two years for which SES data was collected (Supplementary Table S2). In 2008, coverage of all three intended courses of SMC in the poorest SES quintile was estimated at 94.5%, which was higher than all but the highest SES quintile, which achieved 94.9% coverage. In 2009, all coverage levels dropped across all quintiles, but the 85.3% coverage achieved in the poorest quintile was only slightly lower than the 87.5% coverage in the highest quintile.

**Figure 4:**
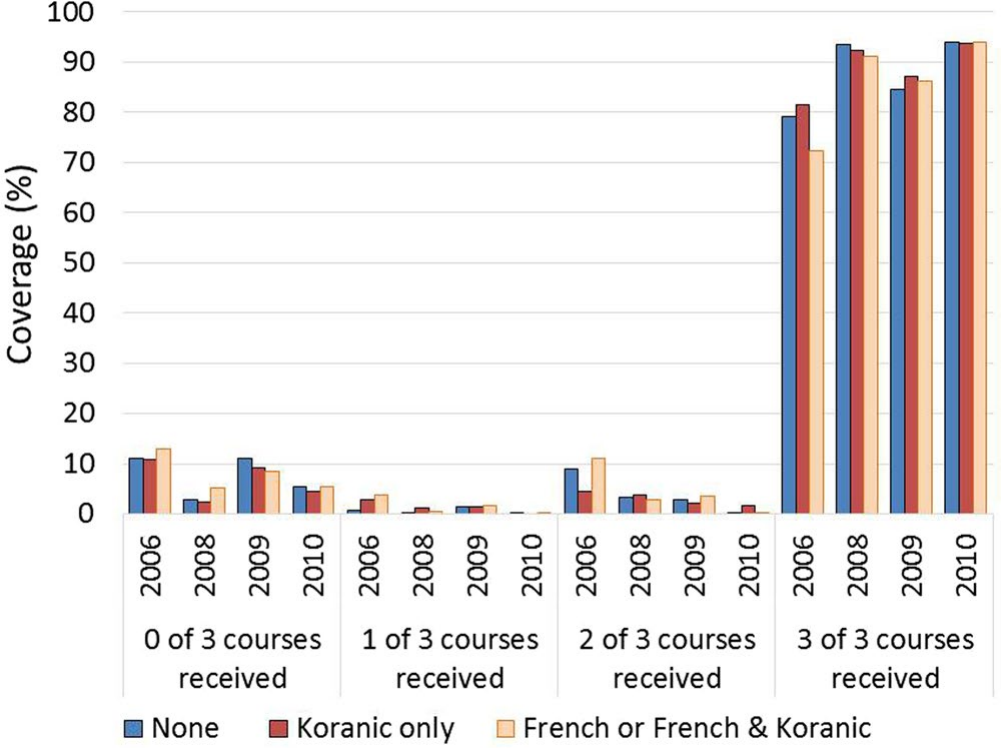
Equity of SMC coverage by mother’s education in 2008, 2009, and 2010 The figure presents the proportion of children targeted to receive SMC who received 0, 1, 2, or 3 of the 3 intended courses of treatment each year, disaggregated by whether the child’s mother had no education, some Koranic education only, or some French or French and Koranic education.

Coverage of SMC was also equitable with respect to the education of the child’s mother. Figure 4 illustrates that there was no evidence in 2008 (p = 0.55), 2009 (p = 0.77), or 2010 (p = 0.33) of differences in coverage according to whether the mother had no education, only a Koranic education, or any French education (Supplementary Table S3). In all three years, coverage levels were very similar between groups, and in both 2008 and 2010, children whose mothers had no education achieved the highest levels of SMC coverage.

This equitable and very high coverage of SMC contrasts sharply with the inequitable and lower rates of use of bed nets shown in Fig. 3. In both years analysed, there was very strong evidence of a regressive trend in those children who had slept under a long-lasting insecticide-treated net (LLIN), any treated net, or any net in the night preceding the survey (Supplementary Table S2). In 2008, use of an LLIN in the night preceding the survey varied from 54% in the poorest SES quintile to 69% in the highest SES quintile, reflecting an 18% (8% to 29%) increase in the odds of LLIN use for each level increase in SES quintile. In 2009, the inequity in bed net usage became even starker as only 39% of children in the poorest quintile had slept under an LLIN the preceding night compared with 75% of children in the highest SES quintile. The odds ratio for the increase in coverage corresponding to an increase of one level in socioeconomic ranking, was 1.0 (95% CI 0.88, 1.2) for receiving 3 SMC treatments in 2008, and 1.1 (0.95, 1.2) in 2009, compared with odds ratios of 1.2 (1.1, 1.3) and 1.3 (1.2, 1.5) for sleeping under an LLIN in 2008 and 2009 respectively.

## Discussion

This study demonstrated that SMC could be effectively delivered on a large scale through routine health services using a door-to-door approach, achieving high and equitable coverage, with good adherence to supervised and unsupervised daily treatment doses. Over 5 years, implementation was gradually scaled up from pilot delivery of SMC to more than 5,000 children in 3 health posts in 2006 to large-scale delivery to more than 150,000 children in the catchment areas of 46 health posts in 2010. The coverage of SMC achieved was consistently higher than and more equitable than that of bednet use, indicating that SMC reached children who were not protected by bed nets, and the strategy successfully reached older school-age children. These findings strengthen the case for implementing SMC door-to-door, and for including older children.

Coverage was defined in terms of the number of treatments received and therefore did not capture SMC contacts where the child was referred to the clinic and did not receive SMC treatment at the clinic. An alternative definition of coverage, counting all contacts where the guidelines were followed, could also be defined. SMC record cards would need to be modified to capture such contacts.

Findings from this study contributed to the policy recommendation for SMC^1^ and the field guide for SMC^10^. The WHO guide should be consulted for the currently recommended approach to SMC administration. Separate tablets for infants and children are now available in blister packs, avoiding the need to break tablets, in dispersible formulations.

It had been questioned whether SMC could be delivered on a large scale in the absence of an established delivery route for reaching older children once a month. The demonstration of feasibility and acceptability on a large scale was therefore a necessary step before a policy recommendation^1^ could be made. The size of the study (which was originally motivated by power calculations to measure the effect on mortality), permitted piloting of delivery on a large scale. The study was also able to demonstrate that good compliance with monthly treatments and adherence to daily doses could be achieved through a door-to-door approach with supervision of the first dose and the remaining doses being administered by the caregiver.

Engagement between researchers and health staff at all levels during the planning phase was a key step in choosing a suitable delivery approach and in planning the evaluation. The study also benefitted from international consultations between researchers involved in SMC and policy makers, which were organised independently of this study, a meeting in Dakar in 2008 and a meeting with WHO GMP in early 2010 which led to a refinement of the delivery approach and additional data collection to allow more detailed assessment of safety and costs.

In 2011, the Technical Expert Group on Chemoprevention met to review evidence on SMC^11^. One recommendation of this group was that SMC should be prioritised in areas where the incidence of malaria was at last 0.1 episodes per child during the main transmission period as it was expected that the intervention would be highly cost effective in such circumstances. The study area fell outside this recommendation, but since 2013, Senegal’s National Malaria Control Programme in Senegal has implemented SMC in the southern regions of the country, where transmission is more intense. In 2017, twelve countries in the Sahel and sub-Sahel, including Senegal, have SMC programmes. In Senegal, the decision was taken to include children up to 10 years of age in the national SMC programme. Our study has shown that school-age children can be effectively reached and the time for delivery was not greatly increased by including these children.

A door-to-door approach was employed in this study. Experience with different modes of delivery is being gained^12,13^ and evaluations are being undertaken through the ACCESS-SMC project^14^. Delivery through fixed points has been less effective than delivery door to door. In a pilot project in Mali^13^, SMC was delivered at a central point in each village, or at the health centre, the first daily dose administered by health workers and the remaining doses left with the caregiver. Coverage was lower than in our study (average coverage of first doses was 77% over 4 cycles, and 54% received treatment at all 4 cycles).

Door-to-door approaches are also used for polio campaigns, biannual health days for Vitamin A and deworming, and LLIN campaigns and azithromycin campaigns for trachoma, but few evaluations of the delivery approach have been published. Combining SMC with Vitamin A delivery was piloted in this study in three health posts. The complication of different dosing by age necessitated the presence of an additional person in each CHW delivery team. A larger evaluation had been planned but the supply chain and finance for Vitamin A is coordinated independently of the malaria control programme and synchronisation of SMC and Vitamin A delivery was problematic in practice. Combining SMC with community case management has been shown to offer substantial advantages^15^. Combining SMC with other strategies, including nutritional screening, requires further evaluation.

Our study offers several practical insights for implementation research. While we designated the first two years as “the pilot study”, the delivery strategy continued to be adapted, with changes to the delivery strategy in all three years of the “large-scale implementation”. Modifications were made to improve the programme, to respond to problems that arose, for example with suppliers, and to move towards a delivery model in which researchers played little or no role. In our study, SMC courses were recorded in a register and on the mother’s card. Accurate recording of courses received is essential if impact is to be monitored, using, for example, case control studies to measure efficacy of the intervention or rebound effects, and for adverse event monitoring. Publicity campaigns and other sensitization activities played an important role in ensuring good uptake, and weaknesses in this area likely explain the dip in coverage and the high rate of absences in 2009, which was remedied in 2010. We have identified a number of practical lessons for large-scale implementation, including the importance of engagement between researchers and health staff at all levels during planning, and the value of piloting and gradual scale-up. High coverage was achieved in older, school-aged children, by informing community leaders and school teachers about dates of SMC administration, and by delivering the treatment on Friday afternoons and weekends.

This was the first study to evaluate implementation effectiveness of SMC under operational conditions, and to assess the feasibility of reaching older children. Robust methods were used for estimation of coverage and equity. Age-specific tablet strengths designed and packaged for SMC, which are now available, were not available at the time the study was conducted. These new products may reduce administration errors and permit more precise dosing as they avoid the need to break tablets, and may promote adherence. However, highly effective delivery was possible with the single-strength loose tablets used in the study. Involvement of researchers may have contributed to implementation effectiveness but this involvement was limited and reduced over the curse of the implementation. Quantification of drug requirements benefited from the existence of the DSS system and listings of children may have helped correctly to identify eligible children and their dose.

## Conclusion

Seasonal malaria chemoprevention can be delivered on a large scale through routine health services and achieve high and equitable coverage both amongst children under 5 and amongst children aged 5 to 9 years. The existence of a well-functioning CHW network and wider primary health care system, close collaboration with local health workers from the start, and gradual scale-up and adaptation of intervention methods all contributed to the success of implementation. Our experience offers several practical lessons both for those implementing SMC and those considering other campaigns delivered through community health workers. Delivering SMC to school-age children is feasible and the results of this study should encourage countries with SMC programmes to consider including older children in SMC programmes.

### Data availability statement

Surveillance System data, and individual-level survey data, are available at http://dx.doi.org/10.17037/DATA.117. Requests for access will be reviewed by a Data Access Committee to ensure use of the data protects participant privacy according to the terms of participant consent and ethics committee approval.

## Electronic supplementary material

Supplementary information
