# Author Correction: Implementation, coverage and equity of large-scale door-to-door delivery of Seasonal Malaria Chemoprevention (SMC) to children under 10 in Senegal

**DOI:** 10.1038/s41598-018-25426-4

**Published:** 2018-05-01

**Authors:** El-Hadj Bâ, Catherine Pitt, Yankhoba Dial, Sylvain Landry Faye, Matt Cairns, Ernest Faye, Mouhamed Ndiaye, Jules-Francois Gomis, Babacar Faye, Jean Louis Ndiaye, Cheikh Sokhna, Oumar Gaye, Badara Cissé, Paul Milligan

**Affiliations:** 1 Institut de Recherche pour le Développement, Dakar, Senegal; 2 London School of Hygiene & Tropical Medicine, London, UK; 3 Ministère de la Santé et de la Prévention, Dakar, Senegal; 4 Université Cheikh Anta Diop, Dakar, Senegal

Correction to: *Scientific Reports* 10.1038/s41598-018-23878-2, published online 03 April 2018

The Acknowledgements section in this Article is incomplete.

“We are grateful to the district health teams in Tivaouane, Mbour, Fatick, Bambey and Niakhar districts, to the communities in those districts, and to the project field staff, for their participation. The funders had no role in study design, data collection and analysis, decision to publish, or preparation of the manuscript.”

should read:

“We are grateful to the district health teams in Tivaouane, Mbour, Fatick, Bambey and Niakhar districts, to the communities in those districts, and to the project field staff, for their participation. The funders had no role in study design, data collection and analysis, decision to publish, or preparation of the manuscript. MC acknowledges funding from an MRC Population Health Scientist Fellowship (ref: MR/J012394/1). This award is jointly funded by the UK Medical Research Council (MRC) and the UK Department for International Development (DFID) under the MRC/DFID Concordat agreement and is also part of the EDCTP2 programme supported by the European Union.”

