# Supplementary material for: Implementation, coverage and equity of large-scale door-to-door delivery of Seasonal Malaria Chemoprevention (SMC) to children under 10 in Senegal
Source: Sci Rep. 2018 Apr 3;8:5489. doi: 10.1038/s41598-018-23878-2 (PMC5882955; doi:10.1038/s41598-018-23878-2)
Supplement: Supplementary file 1 — Supplementary information [file 41598_2018_23878_MOESM1_ESM.doc]

**SUPPLEMENTARY INFORMATION**

**Implementation, coverage and equity of large-scale door-to-door delivery of Seasonal Malaria Chemoprevention (SMC) to children under 10 in Senegal.**

Authors: El-Hadj Bâ, Catherine Pitt, Yankhoba Dial, Sylvain Landry Faye, Matt Cairns, Ernest Faye, Mouhamed Ndiaye, Jules-Francois Gomis, Babacar Faye, Jean Louis Ndiaye, Cheikh Sokhna, Oumar Gaye, Badara Cissé, Paul Milligan

**Table S1. Changes in dosage and formulations by age over time**

**Table S2. Equity: Coverage of SMC compared with bed nets by socio-economic status**

**Table S3. Equity: Coverage by mother's education**

**Table S4: Coverage by month**

**Table S1. Changes in dosage and drug formulations by age over time**

Community health workers (CHWs) were trained to administer a supervised dose of amodiaquine (AQ) and sulphadoxine-pyrimethamine (SP) to children and to provide their caregivers with two further doses of AQ to administer at home on the following two days. The correct dose was determined by age range and for the youngest and oldest children required splitting tablets.

|  |  |  | Number of tablets received per day (Number of days) | | | | | | Formulation | |  |
| --- | --- | --- | --- | --- | --- | --- | --- | --- | --- | --- | --- |
|  |  | Age group | < 24 months^1^ | | 24 - 72 months^2^ | | 72 - 120 months | |  |  |  |
| Phase | Year | Month | SP (x1d) | AQ (x3d) | SP (x1d) | AQ (x3d) | SP (x1d) | AQ (x3d) | SP | AQ |  |
| Pilot | 2006 | September | 0.5 | 0.5 | 1 | 1 | NA | NA | 500mg sulfamethoxypirazyne (sulfalene)/ 25mg pyrimethamine | 200mg | Breakable, non-dispersible tablets |
|  |  | October | 0.5 | 0.5 | 1 | 1 | NA | NA | 500mg sulfalene /25mg pyrimethamine | 200mg | Breakable, non-dispersible tablets |
|  |  | November | 0.5 | 0.5 | 1 | 1 | NA | NA | 500mg sulfalene /25mg pyrimethamine | 200mg | Breakable, non-dispersible tablets |
|  | 2007 | September | 0.5 | 0.5 | 1 | 1 | NA | NA | 500mg sulfalene /25mg pyrimethamine | 200mg | Breakable, non-dispersible tablets |
|  |  | October | 0.5 | 0.5 | 1 | 1 | NA | NA | 500mg sulfalene /25mg pyrimethamine | 200mg | Breakable, non-dispersible tablets |
|  |  | November | 0.5 | 0.5 | 1 | 1 | NA | NA | 500mg sulfalene /25mg pyrimethamine | 200mg | Breakable, non-dispersible tablets |
| Main study | 2008 | September | 0.5 | 0.5 | 1 | 1 | NA | NA | 500mg sulfalene /25mg pyrimethamine | 200mg | Breakable, non-dispersible tablets |
|  |  | October | 0.5 | 0.5 | 1 | 1 | NA | NA | 500mg sulfalene /25mg pyrimethamine | 200mg | Breakable, non-dispersible tablets |
|  |  | November | 0.5 | 0.5 | 1 | 1 | NA | NA | 500mg sulfalene /25mg pyrimethamine | 200mg | Breakable, non-dispersible tablets |
|  | 2009 | September | 0.5 | 0.5 | 1 | 1 | 1.5 | 1.5 | 500mg sulphadoxine/ 25mg pyrimethamine | 153mg | Non-breakable, non-dispersible tablets |
|  |  | October | 0.5 | 0.5 | 1 | 1 | 1.5 | 1.5 | 500mg sulphadoxine/ 25mg pyrimethamine | 153mg | Non-breakable, non-dispersible tablets |
|  |  | November | 0.5 | 0.5 | 1 | 1 | 1.5 | 1.5 | 500mg sulphadoxine/ 25mg pyrimethamine | 153mg | Non-breakable, non-dispersible tablets |
|  | 2010 | September | 0.5 | 0.5 | 1 | 1 | 1.5 | 1.5 | 500mg sulphadoxine/ 25mg pyrimethamine | 153mg | Non-breakable, non-dispersible tablets |
|  |  | October | 0.5 | 0.5 | 1 | 1 | 1.5 | 1 | 500mg sulphadoxine/ 25mg pyrimethamine | 200mg | Breakable, dispersible (AQ also sweetened) |
|  |  | November | 0.5 | 0.5 | 1 | 1 | 1.5 | 1 | 500mg sulphadoxine/ 25mg pyrimethamine | 200mg | Breakable, dispersible (AQ also sweetened) |
| ^1^In 2006 and 2007, children were eligible from the age of 2 months; from 2008 onwards, only children aged from 3 months were eligible.  ^2^note that only children <60 months were eligible until 2009. | | | | | | | | | | | |

**Table S2. Equity: Coverage of SMC compared with bed nets by socio-economic status**

Denominators (N) refer to SMC implementation areas. LLIN: Long-lasting insecticide-treated bed net.

|  |  | **Socio-economic status (SES) quintile** | | | | |  |  |
| --- | --- | --- | --- | --- | --- | --- | --- | --- |
| **2008** |  | **Lower** | **Lower middle** | **Middle** | **Upper middle** | **Upper** | **Odds ratio for one level increase in SES** | **P-value for trend** |
|  | N | 253 | 154 | 171 | 190 | 251 |  |  |
|  | Received all 3 SMC courses (%) | 94.5 | 91.2 | 87.6 | 90.1 | 94.9 | 1.04 (0.88, 1.22) | 0.63 |
|  | Slept under any net last night (%) ^e^ | 69.9 | 74.4 | 73.1 | 86.5 | 82.0 | 1.22 (1.04, 1.42) | 0.012 |
|  | Slept under LLIN last night (%) ^f^ | 53.3 | 53.9 | 58.3 | 68.1 | 67.9 | 1.19 (1.07, 1.33) | 0.002 |
|  | Slept under treated or impregnated net or LLIN last night (%) ^g^ | 64.9 | 68.6 | 69.6 | 76.8 | 70.3 | 1.08 (0.95, 1.21) | 0.225 |
| **2009** | N | 689 | 725 | 631 | 674 | 678 |  |  |
|  | Received all 3 SMC courses (%) | 85.3 | 80.0 | 86.4 | 83.4 | 87.5 | 1.07 (0.95, 1.20) | 0.36 |
|  | Slept under any net last night (%) ^e^ | 42.9 | 60.9 | 55.6 | 61.5 | 77.2 | 1.34 (1.18, 1.53) | <0.001 |
|  | Slept under LLIN last night (%) ^f^ | 39.9 | 57.3 | 53.0 | 56.0 | 74.7 | 1.33 (1.18, 1.50) | <0.001 |
|  | Slept under treated or impregnated net or LLIN last night (%) ^g^ | 42.4 | 59.6 | 55.3 | 60.7 | 76.4 | 1.34 (1.18, 1.52) | <0.001 |

**Table S3. Equity: Coverage by mother's education**

French: French school and Koranic school or French school only.

|  | **Year** | **2008** | | | **2009** | | | **2010** | | |
| --- | --- | --- | --- | --- | --- | --- | --- | --- | --- | --- |
|  | Level/ Type of education | **None**  **(N=586)** | **Koranic only**  **(N=152)** | **French**  **(N=235)** | **None**  **(N = 1916)** | **Koranic only**  **(N = 608)** | **French**  **(N = 699)** | **None**  **(N = 502)** | **Koranic only**  **(N = 141)** | **French**  **(N = 195)** |
| **Number of courses** | 0 | 2.9 | 2.5 | 5.2 | 11.1 | 9.3 | 8.6 | 5.6 | 4.5 | 5.5 |
|  | 1 | 0.4 | 1.2 | 0.6 | 1.4 | 1.4 | 1.7 | 0.12 | 0 | 0.34 |
|  | 2 | 3.3 | 3.9 | 3.0 | 3.0 | 2.2 | 3.5 | 0.24 | 1.65 | 0.34 |
|  | 3 | 93.4 | 92.4 | 91.2 | 84.5 | 87.1 | 86.2 | 94.0 | 93.8 | 93.9 |
| **Design-based p-value for test of difference between groups** |  | 0.55 |  |  | 0.77 |  |  | 0.33 |  |  |

**Table S4: Coverage by month 2008-2010 (with 95%CI)**

|  | **<5yrs** |  |  | **5-9yrs** |  |  |
| --- | --- | --- | --- | --- | --- | --- |
|  | **September** | **October** | **November** | **September** | **October** | **November** |
| **2008** | 0.95 (0.93,0.97) | 0.95 (0.93,0.97) | 0.93 (0.91,0.96) |  |  |  |
| **2009** | 0.86 (0.83,0.88) | 0.84 (0.82,0.87) | 0.83 (0.80,0.86) | 0.89 (0.87,0.91) | 0.90 (0.87,0.92) | 0.89 (0.86,0.91) |
| **2010** | 0.91 (0.87,0.95) | 0.91 (0.87,0.95) | 0.91 (0.87,0.95) | 0.97 (0.95,0.98) | 0.97 (0.95,0.99) | 0.97 (0.95,0.98) |
